# Enhanced NH_3_-Sensitivity of Reduced Graphene Oxide Modified by Tetra-α-Iso-Pentyloxymetallophthalocyanine Derivatives

**DOI:** 10.1186/s11671-015-1072-3

**Published:** 2015-09-24

**Authors:** Xiaocheng Li, Bin Wang, Xiaolin Wang, Xiaoqing Zhou, Zhimin Chen, Chunying He, Zheying Yu, Yiqun Wu

**Affiliations:** Key Laboratory of Functional Inorganic Material Chemistry, Ministry of Education, School of Chemistry and Materials Science, Heilongjiang University, Harbin, 150080 China; Shanghai Institute of Optics and Fine Mechanics, Chinese Academy of Sciences, P.O. Box 800216, Shanghai, 201800 China; Heilongjiang Institute of Technology, Harbin, 150050 China

**Keywords:** Phthalocyanine, Reduced graphene oxide, Hybrid, Ammonia, Gas sensor

## Abstract

Three kinds of novel hybrid materials were prepared by noncovalent functionalized reduced graphene oxide (rGO) with tetra-α-iso-pentyloxyphthalocyanine copper (CuPc), tetra-α-iso-pentyloxyphthalocyanine nickel (NiPc) and tetra-α-iso-pentyloxyphthalocyanine lead (PbPc) and characterized by Fourier transform infrared spectroscopy (FT-IR), ultraviolet–visible spectroscopy (UV–vis), Raman spectra, X-ray photoelectron spectroscopy (XPS), transmission electron microscope (TEM), and atomic force microscope (AFM). The as-synthesized MPc/rGO hybrids show excellent NH_3_ gas-sensing performance with high response value and fast recovery time compared with bare rGO. The enhancement of the sensing response is mainly attributed to the synergism of gas adsorption of MPc to NH_3_ gas and conducting network of rGO with greater electron transfer efficiency. Strategies for combining the good properties of rGO and MPc derivatives will open new opportunities for preparing and designing highly efficient rGO chemiresistive gas-sensing hybrid materials for potential applications in gas sensor field.

## Background

It is well known that the carbon nanotubes (CNTs) are considered as an excellent candidate for gas-sensing applications, due to unique electronic and structural characteristics of CNTs. Lots of research results have been reported on the CNT materials used as gas-sensing devices and show excellent gas-sensing properties toward NH_3_, NO, CO, CH_4_, and NO_2_ [1, 2]. As a material similar to CNTs, graphene and reduced graphene oxide (rGO) are the novel nanoscaled materials, atomic-thick layer of sp^2^-bonded carbon atoms, which has two-dimensional honeycomb nanostructure. Because of its unique nanostructure, graphene has exceptional mechanical, thermal, and electrical properties, high electron mobility, and outstanding conductivity at room temperature [3, 4]. These properties make it promising as one of the most appealing carbon materials for electrochemical devices, optoelectronic devices, and sensing devices.

RGO has been demonstrated as a promising chemical-sensing material because of the following merits [5, 6]. Firstly, rGO has a large specific surface area. All atoms of rGO sheet can be considered as surface atoms, and they are capable of absorbing gas molecules, which are a benefit for the charge transfer and provide an enough sensing area for gas adsorption and desorption [7]. Secondly, its inherently low electric noise which makes the charge transfer is more stable than 1D structure such as CNTs. Moreover, rGO possesses the most advantages of CNTs and low price, which makes rGO more suitable for the large-scale research and application. RGO has shown excellent performance for detecting NH_3_, NO_2_, NO, and warfare and explosive agents [6, 8–10]. For instance, Fowler et al. [8] reported the development of useful rGO sensors from chemically converted graphene for NO_2_, NH_3_, and 2, 4-dinitrotoluene. Hu et al. presented a useful gas sensor based on chemically rGO, which can be used as an excellent sensing material and shows excellent responsive repeatability to DMMP [9]. Lu et al. demonstrated high-performance gas sensors based on partially reduced graphene oxide (rGO) sheets via low-temperature thermal treatments [10]. They observed that the rGO showed p-type semiconducting behavior in ambient conditions and was responsive to low-concentration NO_2_ and NH_3_ gases diluted in air at room temperature.

However, there are many problems about the development of rGO-based sensors, such as the slow recovery time, poor selectivity, less-than-ideal solubility, and half-baked film method. Functionalized modifications play more and more important roles in improving the sensing performance of rGO-based sensors, due to their synergetic combination of rGO and functionalized molecules [11], such as polymers [12], metals [13], and metal oxides [14]. A flourishing research area is focused on the functionalization of rGO with metalophthalocyanine (MPc) complexes for enhancing the electronic, optical, and sensing properties [15, 16]. However, few reports have been concentrated on the application of the MPc/rGO hybrids toward the development of gas sensors.

MPc has been widely studied as an excellent sensing material due to the following virtues [17, 18]. First of all, it has high sensitivities as well as great thermal and chemical stability. In addition, MPc includes a planar *π*-conjugated skeleton and adjustable structure. Its center metal, peripheral, and axial substituent group can be changed, which provides possibilities for us to design the target hybrids according to our ideas. More importantly, the peripheral active ground of MPc and the in-plane system with a large *π*-conjugated structure provide a profitable tendency to combine the large basal plane of carbon materials through noncovalent or covalent modification and improve the target hybrids’ gas-sensing properties.

Functionalization of CNTs with MPc has been extensively studied in many groups and exhibits admirable sensing properties, which revealed that MPc plays important roles in enhancing the sensing performance of CNT-based gas sensors [1, 2]. However, MPc can not easily stably grow on 1D CNTs, due to their high curvature. Herein, we developed a method to evenly load the MPc on rGO and MPc/rGO hybrid gas sensors. The key to this method is to choose tetra-α-iso-pentyloxyphthalocyanine copper, nickel, and lead with excellent solubility, large *π*-conjugate system, and superior NH_3_-sensing performance in the previous studies [18]. MPc molecules have been successfully anchored on the surface of rGO sheets by noncovalent *π*–*π* stackable interaction. The resultant MPc/rGO hybrids exhibited prominent gas-sensing response and fast recovery performance. And the sensing mechanism was also deduced in this paper.

## Methods

**Fig. 1:**
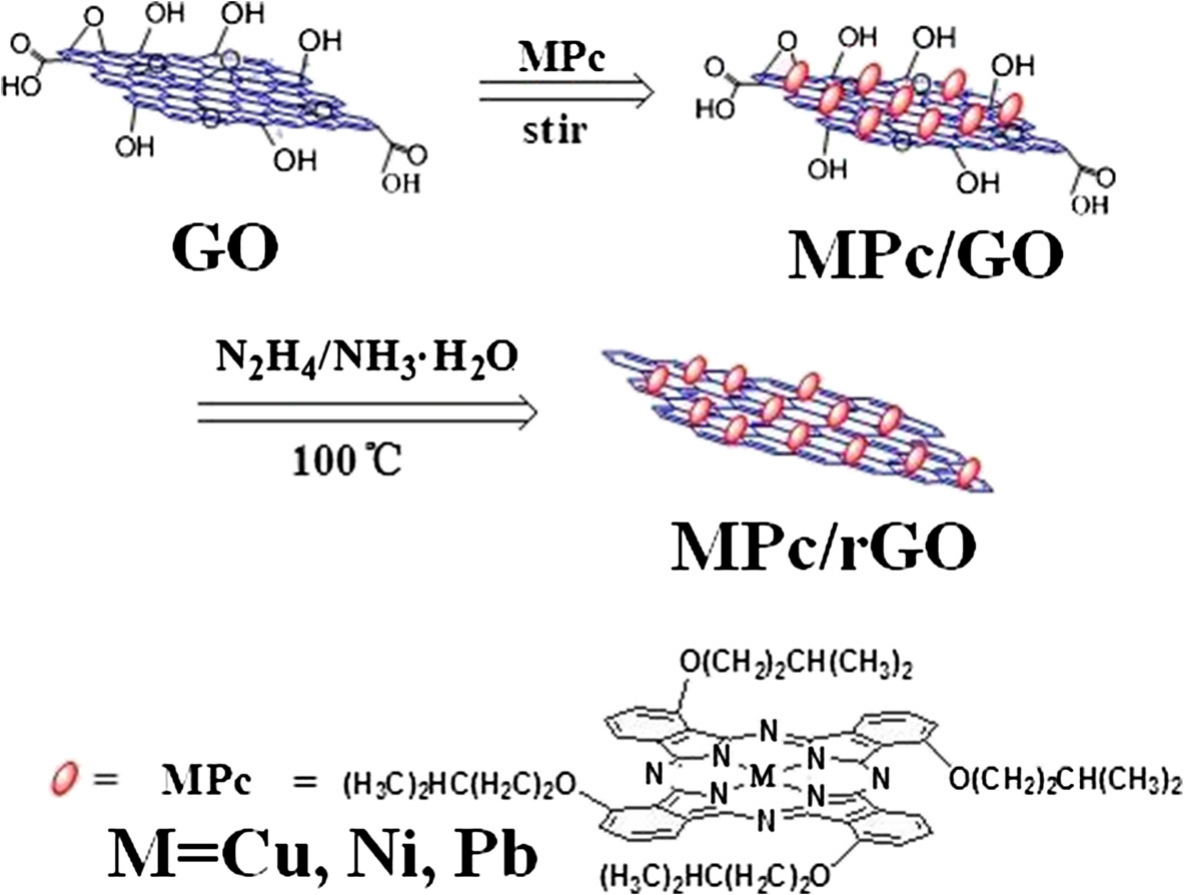
The schematic interaction process for the preparation of MPc/rGO derivatives hybrids

The MPc derivatives were already synthesized on the basis of an established procedure [19]. Flake graphite powder was purchased from Shenzhen Nanotech PortCo. Ltd. All other materials were obtained from Tianjin Fuyu Fine chemical Co., Ltd. and used without further purification. Double distilled deionized water was used with further purification by Millipore Milli-Q system. Graphene oxide (GO) was prepared using the modified Hummers method from graphite powder, which was described in our previous reports [20]. To prepare MPc/rGO hybrids, the hydrazine reduction of GO in the presence of MPc was adopted. The schematic interaction process for the preparation of MPc/rGO hybrids is illustrated in Fig. 1. Simply, the prepared GO was sonicated in the solvent (DMF: H_2_O = 9:1), then the MPc DMF solution was added in the above solvent. The mixture was sonicated for 2 h in the dark about and stirred 24 h. After that, hydrazine and ammonia water were added and stirred at 100 °C for 24 h. The solution was then filtrated and rinsed with CHCl_2_. Finally, the hybrid materials were dried in a vacuum for 24 h. As a comparison, hydrazine and ammonia water were added in the GO solution without MPc and stirred at 100 °C for 2 h. After washing with ethanol and H_2_O, the final product was collected and marked as rGO.

### Structure Characterization

Fourier transform infrared (FT-IR) spectra were recorded on a PE instruments Spectrum One FT-IR spectrometer using the KBr pellet method in the range of 500–4000 cm^−1^. Raman spectroscopy was recorded on a JobinYvon HR800 Raman Spectrometer with excitation from the 450-nm laser source. Ultraviolet–visible (UV–vis) spectra were conducted on a Perkin–Elmer Lambda 900 UV/VIS/NIR spectrophotometer. X-ray photoelectron spectra (XPS) measurements were carried out on a Kratos AXIS Ultra DLD system using monochromated Al Kα X-ray source (1486.6 eV). The morphology and microstructure of the products were characterized using a transmission electron microscope (TEM) with a JEM 2100 instrument at 200 kV utilizing a JEOL FasTEM system and scanning electron microscopy (SEM) with a Hitachi S4800. The surface morphology and thickness of the film deposited on silicon wafer was investigated by a tapping-mode atomic force microscope (AFM, Digital instrument Nanoscope IIIa).

### NH_3_-Sensing Test

The sensing device was fabricated by dip-dropping the DMF suspension of MPc/rGO hybrids onto 5 × 5 mm interdigitated electrode using a microsyringe. These electrodes were fabricated using standard lithography technology and made in the basal of Al_2_O_3_ through sputtering the conductor layer (Au), which have been illustrated by us before [2]. The DMF suspensions of MPc/rGO hybrids (0.5 mg/ml) were prepared by dispersing the MPc/rGO hybrids in DMF. The MPc/rGO hybrids suspensions were sonicated at room temperature for 2 h to make sure that the hybrids were dispersed evenly.

The sensor testing was carried out using a homemade gas-sensing measurement system as illustrated in our previous report [2, 20]. NH_3_ (99.9 %) was purchased from Guangming Research and Design Institute of Chemical Industry, PR China. The electrical resistance of the sensors was measured with a CUST · G2 gas-sensing test system (Advanced Sensor Technology Laboratory of Jilin University, China) by applying a constant DC voltage (3 V) and recording the change in resistance passing through the sensor at a 1-s interval by a computer. In a typical sensing measurement procedure at room temperature 23 °C, (i) sensors were placed in a 10-L volume test chamber provided with a two-way stopcock joined with a pump and a trap. The test chamber was first evacuated, (ii) followed by injection of the NH_3_ gas of the required concentration by a micro syringe with the sweeping clean-air through the first inlet for determination of sensor response for 15 min. (iii) Then the second valve was opened and clean-air was passed in the chamber through an air drying cylinder for recovery determination. After the measurement, both inlet valves were closed so that all the test gas was driven away by the running pump. An electric fan was installed and kept the gas uniform and gas output. The sensing performance of as-fabricated MPc/rGO devices was carried out under external environmental condition (i.e., room temperature 23 °C, the relative humidity 50 % RH). The response of sensors upon exposure to NH_3_ was defined as Response (%) = 100 × (△*R*/*R*_a_) = 100 × (*R*_g_ − *R*_a_)/*R*_a_, where *R*_a_ is the resistance of the sensors before exposure to NH_3_ and *R*_g_ is the resistance in the NH_3_/air mixed gas. The response and recovery times of the films were defined as the times needed to reach 90 % of the final resistance.

## Results and Discussion

### Morphology

**Fig. 2:**
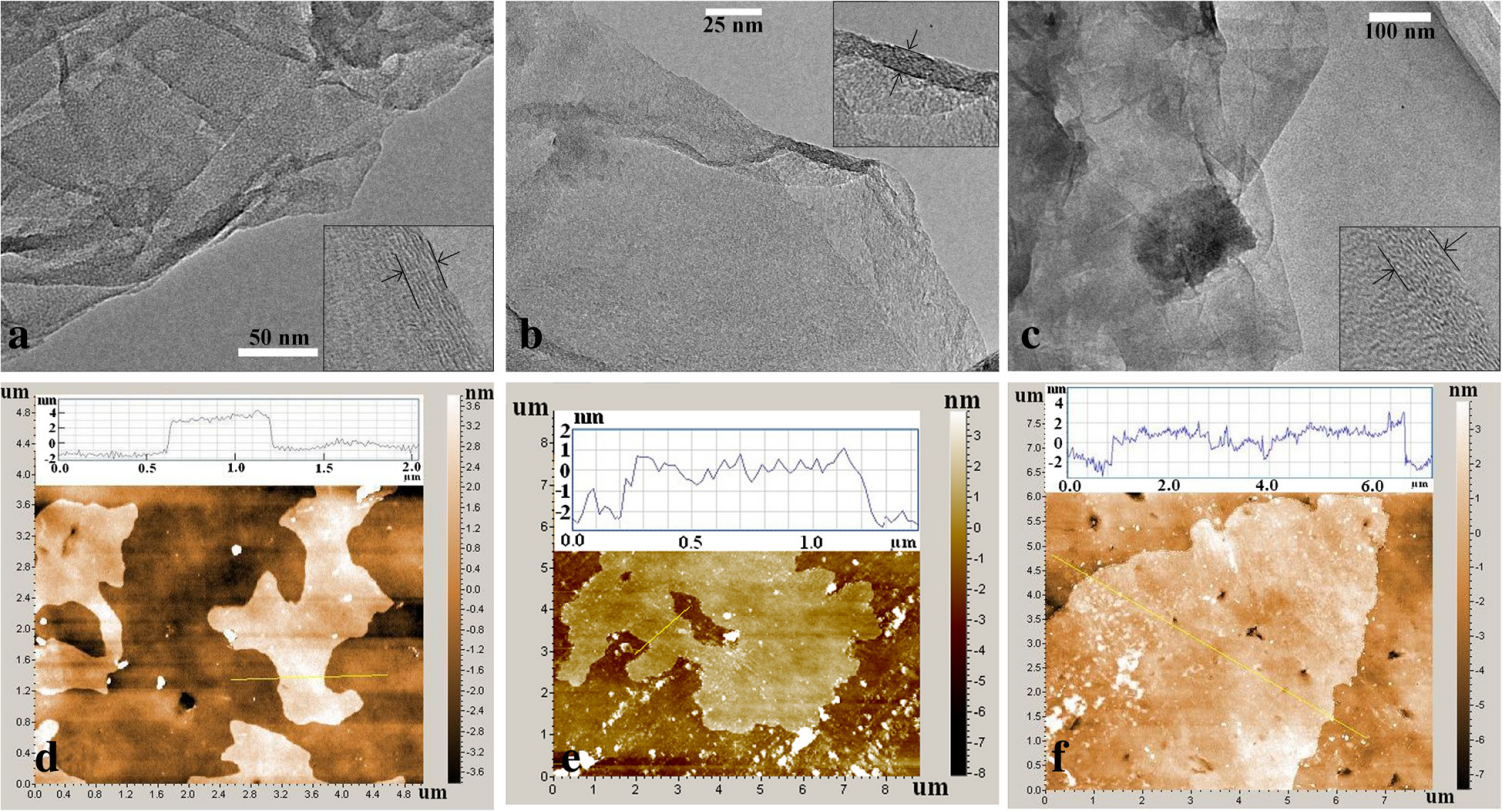
TEM images of the MPc/rGO hybrids: **a** NiPc/rGO, **b** CuPc/rGO, **c** PbPc/rGO. AFM images of the MPc/rGO hybrids: **d** NiPc/rGO, **e** CuPc/rGO, **f** PbPc/rGO, with the *inset* showing height profiles of the sheets

The morphology of MPc/rGO hybrids was analyzed by TEM, as shown in Fig. 2a–c. It was obvious that the MPc/rGO sheets have folds and rolls on its surfaces and formed a blurry rough layer, which indicates that MPc was loaded on surface of rGO. Moreover, the sectional TEM analyses of MPc/rGO hybrids unfold the number of layers of the rGO, which are about ten layers. Figure 2d–f is the atomic force microscope images and section analysis of MPc/rGO deposited on a flat silicon substrate. The AFM images provide detailed information about thin layer of MPc/rGO hybrids sheets. The inset in Fig. 2d–f shows the topographic height profiles for MPc/rGO hybrid sheets. The cross-sectional analysis indicates the thickness of the MPc/rGO hybrids sheets are about 3 nm, which implies also that the hybrid was comprised of multilayered rGO. The thin structure of the MPc/rGO hybrids sheets can increase the absorbed areas, which is very useful for the gas-sensing process.

### UV–vis Absorption Spectra

**Fig. 3:**
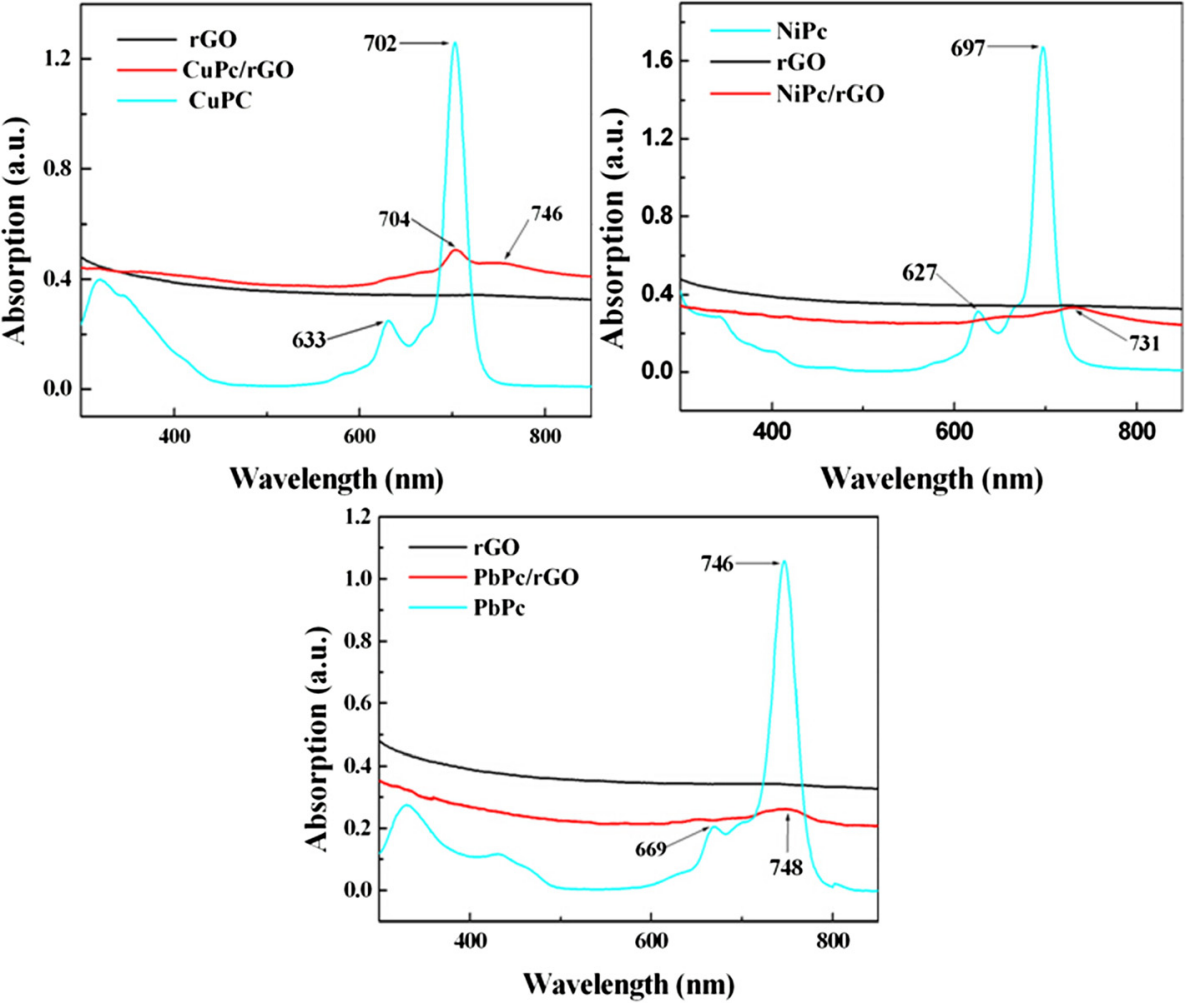
UV–vis spectra of MPc, rGO, and MPc/rGO hybrids dispersed in DMF

The attachment of the small MPc molecules to rGO sheets was supported by the spectroscopic studies using UV–vis spectroscopy. The UV–vis absorption spectra of MPc, rGO, and MPc/rGO in DMF solutions are given and compared in Fig. 3. In the DMF dispersion, the MPc/rGO hybrids showed typical electronic absorption spectra with distinct absorption regions in the visible region at 600–800 nm (Q band), which are corresponding to the absorption peak of the MPc molecules (Q band) [18]. After the noncovalent functionalization, the intensity of the peaks decreased apparently along with the absorption peak red-shifted and become broader for the resultant hybrids comparing with MPc complexes [18], suggesting that the strong *π*–*π* interaction between the rGO and MPc molecules.

### FT-IR Spectra

**Fig. 4:**
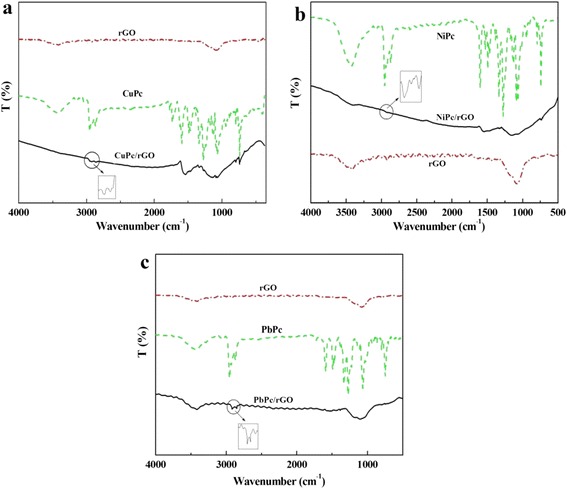
FT-IR spectra of (**a**) rGO, CuPc and CuPc/rGO hybrids; (**b**) rGO, NiPc and NiPc/rGO hybrids; (**c**) rGO, PbPc and PbPc/rGO hybrids

The attachment of the small MPc molecules to rGO sheets was confirmed further by using FT-IR spectroscopy. Figure 4 shows the FT-IR spectra of rGO, MPc, and MPc/rGO hybrids. The typical peaks of rGO located at 3421 and 1068 cm^−1^, which correspond to the O–H stretching and vibration mode of absorbed water. As for the MPc/rGO hybrids, after hybridization, the characteristic peaks of MPc and rGO are found in MPc/rGO hybrids, such as CuPc/rGO hybrid, as shown in Fig. 4a. The characteristic peaks of CuPc derivatives can be found in hybrid material at 2936, 2863 (C–H stretching vibration), 1539, 1490, 1335 (stretching mode of benzene ring as well as C–N vibration), 1061, 1080 (C–O stretching vibration), and 742 cm^−1^ (–CH_2_CH_2_– in-plane vibration swing), which powerfully demonstrated that CuPc molecules had been successfully decorated on the surface of rGO. The characteristic peaks of NiPc and PbPc in NiPc/rGO and PbPc/rGO hybrids can also be observed in Fig. 4b, c respectively. The results indicate that noncovalent anchoring of MPc molecules onto the rGO sheets through *π*–*π* stacking was obtained.

### Raman Spectrum

**Fig. 5:**
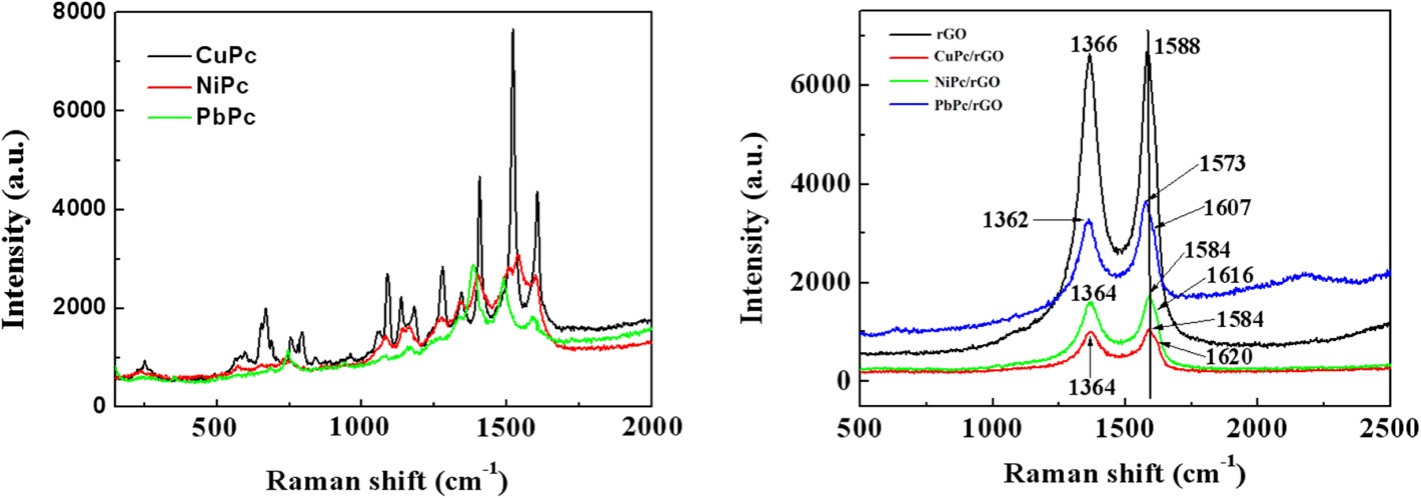
Raman spectra of MPc, rGO, CuPc/rGO, NiPc/rGO, and PbPc/rGO hybrids

The noncovalent adsorption and electron transfer interaction can be also confirmed by Raman spectroscopy. The Raman spectra of MPc, rGO, and MPc/rGO hybrids were shown in Fig. 5. For the rGO sample, the D bands and G bands peaks of rGO could be observed in Fig. 5. The G band at 1588 cm^−1^ is due to the E_2g_ vibrational mode [4] and the D band at 1366 cm^−1^ is a breathing mode [12] of k-point phonons of A_1g_ symmetry. As to the MPc/rGO hybrids, the D bands and G bands were also clearly observed, but a large decrease in the intensity, and the D/G intensity ratios of MPc/rGO (0.863) were same as the D/G intensity ratios of rGO (0.862). Guldi pointed out that the changes of the intensity ratio about the D band to the G band can explain the covalent modification of graphene [21]. So the D/G intensity ratios of MPc/rGO and rGO had no obvious difference, which indicates that the conjugation of rGO was not destroyed and further confirm the noncovalent adsorption. Moreover, a new peak appears at 1620 cm^−1^ in CuPc/rGO hybrids, similarly, the NiPc/rGO and PbPc/rGO hybrids also appear new peaks, which are located at 1616 and 1607 cm^−1^, respectively. The new peaks which appeared in the Raman spectra of MPc/rGO hybrids are mainly due to the peak shift and peak overlapping between the MPc and RGO in the hybrids. In addition, the Raman shift provides important information about the electron transfer interaction between the MPc molecules and the RGO sheets. The G band of CuPc/rGO hybrids appears at 1584 cm^−1^, which is downshifted by 4 cm^−1^ compared to that of rGO (1588 cm^−1^). Similarly, the G band of NiPc/rGO (1584 cm^−1^) and PbPc/rGO (1573 cm^−1^) are downshifted by 4 and 15 cm^−1^, respectively. The shift of G band is attributed to the increased abundance of charge carriers [22] provided by the MPc molecules on the graphene plane, which eventually raises the Fermi level [23].

### XPS Spectrum

**Fig. 6:**
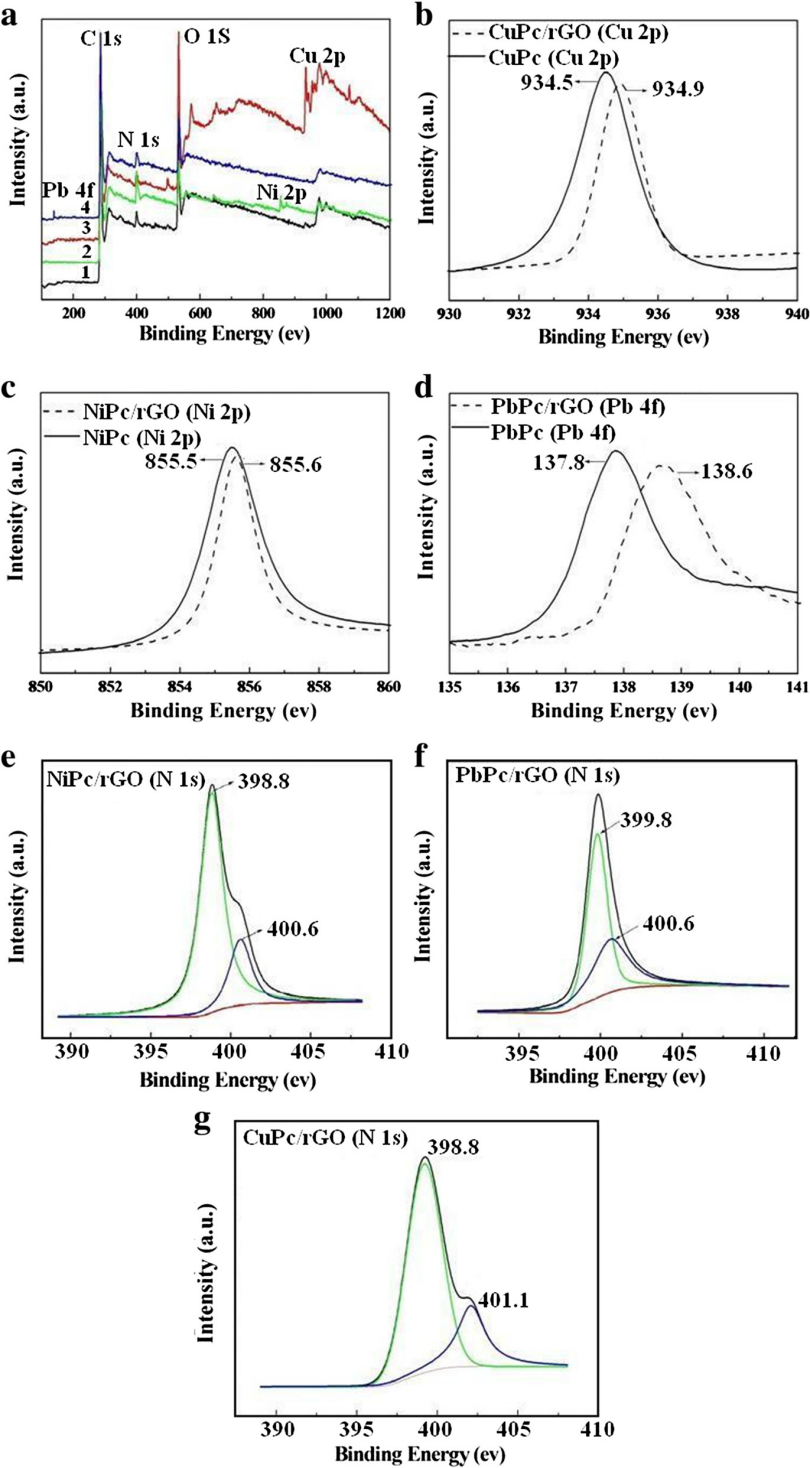
**a** XPS analysis of (*1*) RGO, (*2*) NiPc/rGO, (*3*) CuPc/rGO, and (*4*) PbPc/rGO hybrids survey spectra; **b** Cu 2p XPS spectra of CuPc and CuPc/rGO hybrids, **c** Ni 2p XPS spectra of NiPc and NiPc/rGO hybrids, **d** Pb 4f XPS spectra of PbPc and PbPc/rGO hybrids; N 1s XPS spectra of NiPc/rGO (**e**), CuPc/rGO (**f**), and PbPc/rGO hybrids (**g**)

XPS was also employed to prove the successful attachment of MPc molecules onto the surface of the rGO sheets and demonstrate the charge transfer interaction between MPc molecules and rGO sheets. In the spectra of the hybrids (shown in Fig. 6e–g), the N 1s peaks of MPc and MPc/rGO hybrids consist of two split peaks, which are distributed to two groups of four nitrogen atoms in different chemical environments in the molecules, suggesting the MPc/rGO hybrids have been successfully prepared. As shown in Fig. 6a, all of the MPc/rGO hybrids exhibit the characteristic peaks of C 1s, N 1s, and O 1s. As expected, the appearance of the Cu 2p (934.9 eV), Ni 2p (855.6 eV), and Pb 4f (138.6 eV) peaks corresponding to the spectrum of CuPc/rGO, NiPc/rGO, and PbPc/rGO hybrids further suggests the successful attachment of MPc molecules onto the surface of the rGO sheets. Figure 6b–d show that the Cu 2p peak of the CuPc/rGO hybrid (934.9 eV) upshifts by 0.4 eV compared to that of pure CuPc (Cu 2p peak at 934.5 eV); the Ni 2p peak of the NiPc/rGO hybrid (855.6 eV) upshifts by 0.1 eV compared to that of pure NiPc (Ni 2p peak at 855.5 eV); the Pb 4f peak of the PbPc/rGO hybrid (138.6 eV) upshifts by 0.8 eV compared to that of pure PbPc (Pb 4f peak at 137.8 eV), which also agree with the charge transfer phenomena of the N 1s XPS peaks of the MPc/rGO hybrids shift to higher binding energy compared to that of pure MPc (shown in Fig. 6e–g). These phenomena indicate the charge transfer from MPc molecules to rGO sheets in the hybrids, because the binding energy is correlated to the electron density around the nucleus (the lower the electronic density is, the higher the binding energy). Overall, from the XPS spectra (Fig. 6), we can conclude that MPc molecules were successfully attached onto the surface of the rGO sheets and the electron transfer system was formed from MPc to rGO, which is consistent with the reported electron transfer phenomenon [21].

### Gas-Sensing Properties

**Fig. 7:**
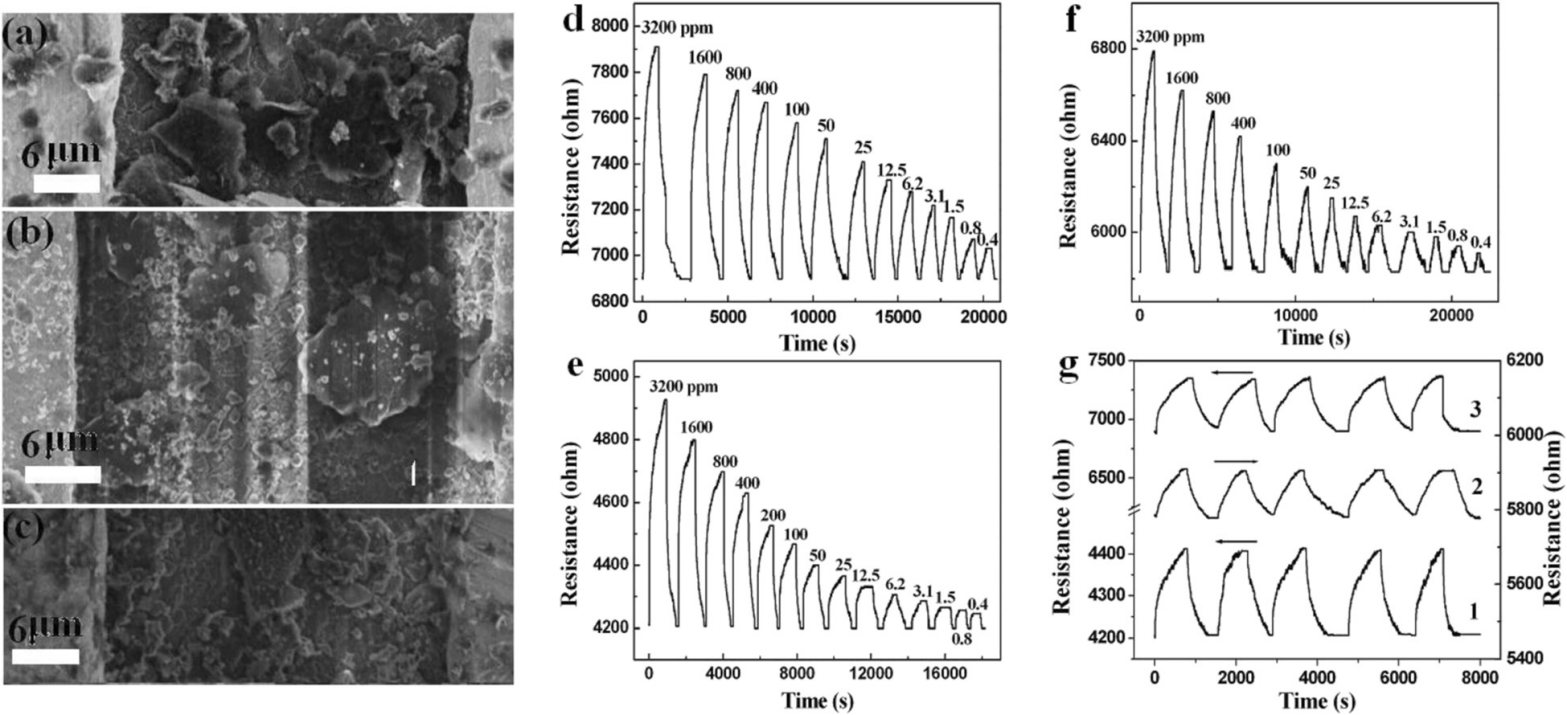
SEM images of **a** CuPc/rGO, **b** NiPc/rGO, and **c** PbPc/rGO hybrids bridged electrode, response, and recovery curves of **d** CuPc/rGO, **e** NiPc/rGO, and **f** PbPc/rGO hybrid sensors in different concentrations of NH_3_ at room temperature and **g** reversibility of the response of (*1*) PbPc/rGO hybrid sensor to 0.75 ppm NH_3_ gas, (*2*) NiPc/rGO hybrid sensor to 50 ppm NH_3_ gas, and (*3*) CuPc/rGO hybrid sensors to 100 ppm NH_3_ gas

The NH_3_ gas-sensing performance of MPc/rGO hybrid sensors were evaluated and compared with sensors made of pure rGO sensor. Sensors were fabricated using a drop casting technique on interdigitated electrode surface. Figure 7a–c shows assembled MPc/rGO hybrids bridging the electrode gaps and the connecting electrodes were formed. The resistance value of 4–7 kΩ was obtained, which suggested that a perfect circuit of the sensing device had been achieved, which is higher than pure rGO sensor (about 2.8 kΩ). Interestingly, the resistance of MPc sensors is remarkably decreased from about 3000 MΩ [18] to 4–7 kΩ, indicating the improved electrical conductivity of MPc/rGO hybrids. This maybe ascribed to the large MPc/rGO hybrids conjugated *π* system and electron transfer interactions from MPc to rGO sheets, which results the increase of the electrical conductivity of MPc and decrease of the electrical conductivity of rGO.

**Fig. 8:**
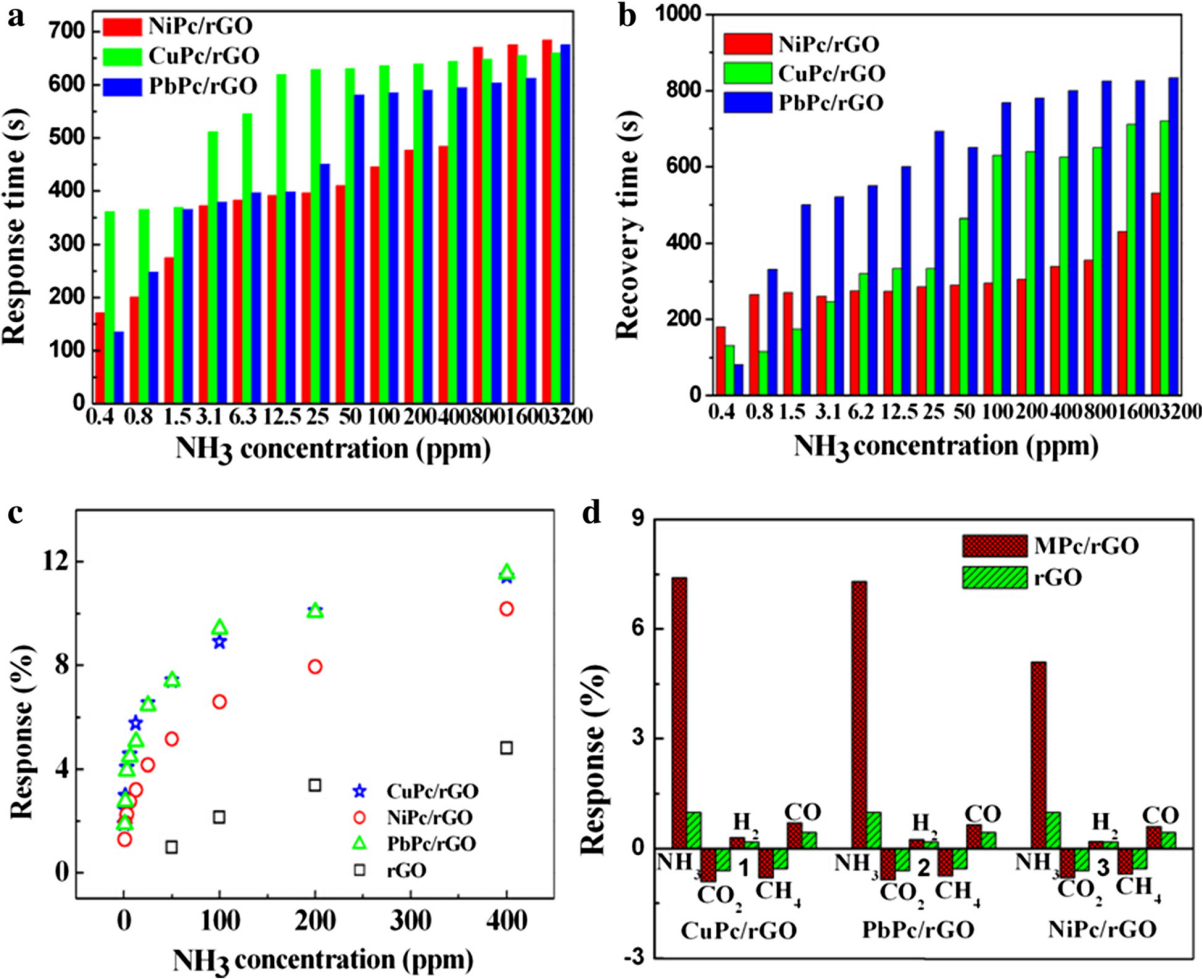
**a** Response times, **b** recovery times, **c** response of rGO and MPc/rGO hybrid sensors versus NH_3_ concentrations at room temperature, **d** response of rGO and MPc/rGO hybrid sensors to 50 ppm NH_3_, CO_2_, H_2_, CH_4_, and CO gas

The resistance trace of sensors was measured with the voltage fixed at 3 V between the interdigitated electrodes. An ohmic response was generated with the MPc/rGO hybrids for NH_3_ gas concentrations of 0.4–3200 ppm, as shown in Fig. 7d–f. The results show that the resistance of the sensors increases dramatically with the increase of NH_3_ gases concentration, indicating the p-type response of MPc/rGO hybrids. The MPc/rGO hybrid sensors recover to the original resistance value in the absence of NH_3_ gases. Figure 8a, b shows response and recovery time of MPc/rGO hybrid sensors to different concentration of NH_3_ gas. For example, the response times of CuPc/rGO, NiPc/rGO, and PbPc/rGO sensors to 800 ppb NH_3_ are 364, 200, 248 s, and the recovery times of CuPc/rGO, NiPc/rGO, and PbPc/rGO sensors to 800 ppb NH_3_ are 115, 264, 331 s, respectively. Generally speaking, the recovery time of MPc/rGO hybrid sensors increases with raised concentration of NH_3_. It is possible that the NH_3_ molecules interact firstly with MPc blending rGO surface, then the NH_3_ molecules move then into the external and inside of rGO, which is more slow recovery process. The bigger the gas concentration, the greater interaction between rGO and NH_3_ gas, the sensors need longer recovery time. But the MPc/rGO hybrid sensors can recover completely to the original value within 830 s, even when the concentration of 3200 ppm is used. As for pure rGO, the resistance of rGO sensor can not recover to the original resistance in an hour with the increase of the NH_3_ gas concentration [20], indicating the improved recovery performance of rGO after their functional modification with MPc. Moreover, the response and recovery time order of MPc/rGO hybrids to NH_3_ coincides with our previous studies of the gas-sensing properties of the individual MPc molecules [18], which indicates that the attachment of MPc plays an important role in gas-sensing performance of MPc/rGO hybrids. The differences of the three kinds of sensors are generally related with the structure of MPc. The order of the radius of metal ions is Cu^2+^ (73 pm) ≈ Ni^2+^ (72 pm) < Pb^2+^ (120 pm), and the number of the d-electron is Pb ^2+^ (10) < Ni^2+^ (8) = Cu^2+^. The smaller the ions radius, the fewer the d-electron, the weaker the d-electron contribution of the central metal to the *π*-electron in conjugated ring, so the acceptor power of phthalocyanine macrocycle is increased, NH_3_ is an electron-donating (reducing) gas, thus strengthening the adsorption with facile production of ionized states and hole traps which are formed from strong interactions from NH_3_ to phthalocyanine macrocycles. Therefore, the recovery time of MPc/rGO hybrids vary between 115 and 331 s.

The response of the sensors as a function of gas concentration is shown in Fig. 8c. For MPc/rGO hybrid sensors, the response to NH_3_ gas is much higher than that of a pure rGO sensor at any corresponding NH_3_ concentration. For CuPc/rGO hybrids, a response of 11.5 % could be observed with the concentration of NH_3_ at 400 ppm. Even in response to as low as 0.8 ppm NH_3_, a response of 2.46 % could be obviously observed. Analogously, for PbPc/rGO and NiPc/rGO hybrids, the responses of 11.4 and 10.2 % could also be observed with the concentration of NH_3_ at 400 ppm, the responses of 1.88 and 1.28 % were still observed for 0.8 ppm NH_3_, respectively. In the meantime, the lowest detectable concentration of the MPc/rGO hybrids sensor is down to 400 ppb NH_3_. But for the pure rGO, only a response of 4.83 % can be observed with the concentration of NH_3_ at 400 ppm. The response of MPc/rGO hybrid sensors to 400 ppm NH_3_ is 2.1–2.4 times than that of pure rGO sensor. The response of MPc/rGO hybrid sensors is more than six times than that of the pure rGO sensor when the NH_3_ gas concentration is decreased to 0.8 ppm. Moreover, the response we got is much better than in the recently reported works on ammonia sensing by carbon nanomaterials [6, 24]. For example, Niu et al. in 2014 got a response of 5.2 % with phosphorus-doped graphene naonosheets in presence of 100 ppm of NH_3_, and Ghosh et al. in 2013 got a response of 5.5 % using chemically reduced graphene oxide in presence of 200 ppm of NH_3_. From these studies, analyses and comparisons between pure rGO and MPc/rGO hybrid sensors, we can draw a conclusion that the MPc/rGO hybrid sensors exhibit prominent response and recovery characteristic to wide ranges of concentration of NH_3_ gases_,_ in particular to lower concentration of NH_3_ gases.

Selectivity is also an important factor for the gas sensors. Therefore, the responses of pure rGO and MPc/rGO hybrid sensors to some interferential gases are shown in Fig. 8d. The sensors show a very weak response to these gases, including CO_2_, CH_4_, H_2_, and CO. It is clearly seen that the MPc/rGO sensors show excellent sensitivity and selectivity to NH_3_. The MPc/rGO hybrids can be considered as an outstanding candidate for gas-sensing applications.

**Fig. 9:**
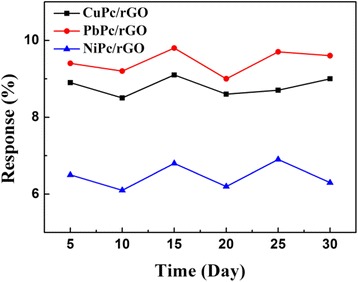
Response of the MPc/rGO hybrid sensors to 100 ppm NH_3_ over long time storage

Stability is also one of the most important characteristics for the sensors. To investigate the time stability of the MPc/rGO hybrid sensors, the sensors were stored in air for subsequent sensing property tests to 100 ppm NH_3_ after the first measurement. The sensors remain the original response and with changes less than 5 % after 30 days (Fig. 9), which indicates that the sensors have a satisfying long-term stability. Moreover, the response changes of repeatability of sensors fabrication were also observed. The response changes of two different MPc/rGO hybrid sensors to 100 ppm NH_3_ over long time storage were also measured. The results indicate the response changes of two different MPc/rGO hybrid sensors are less than 5 % within 30 days. Furthermore, Fig. 7g shows the resistance of the MPc/rGO hybrid sensors as a function of time during exposure to 100 ppm of NH_3_ (five cycles). The result shows that the sensors have good repeatability and no obvious degradation after consecutive measurements. All results indicate that the sensors have an excellent reproducibility, repeatability, and satisfying long-term stability.

The gas mechanism for MPc/rGO hybrid sensors is briefly described as absorbed NH_3_ gas molecules inducing charge transfer interaction. As we known, NH_3_ is a strong electron donor [17], since MPc derivatives are well-known p-type semiconductor, NH_3_ can be chemisorbed on the MPc, which leads to electrons transfer from NH_3_ to MPc. The red shift in UV–vis, binding energy shift in XPS and G band shift in Raman were observed in MPc/rGO hybrids, which powerfully demonstrate the large conjugated *π* system of the hybrids and electron transfer interaction from MPc to rGO sheets. When the MPc derivatives modified on the rGO encounter NH_3_, the transferred electrons from NH_3_ to MPc are easily extracted by rGO. Since rGO is a well-known p-type semiconductor, the electron charge transfer results in a decrease of the hole carrier density, hence causing a marked increase of the resistance. Moreover, the response order of MPc/rGO hybrids to NH_3_ coincides with our previous studies of the gas-sensing properties of the individual MPc molecules [18], which indicates that the attachment of MPc plays an important role in gas-sensing performance of MPc/rGO hybrids. The possible reasons for the improved gas-sensing property of MPc/rGO hybrids are discussed. Firstly, the principle of gas sensing for the resistance-type sensors is based on the conductance variations of the sensing element. Thus, the superior electrical property of rGO contributes to the improved conductivity of hybrids, leading to a better sensing behavior. Secondly, benefited from the existence of rGO, the large surface area facilitates the gas adsorption and diffusion on the active surface [25]. Thirdly, the attachment of MPc derivatives onto the surface of the rGO results in the specific capture and migration of electrons from MPc to rGO. The role of rGO as an electron mediator further facilitates the electron transfer from NH_3_ to MPc molecules. Meanwhile, the four iso-pentyloxy grounds of MPc may donate electrons to the phthalocyanine *π*-system as electron donor grounds, weakening MPc interactions with electron donating NH_3_ and reducing the adsorption between phthalocyanine macrocycles and NH_3_. Therefore, MPc/rGO hybrids exhibit better recoverability than pure rGO by noncovalent modification between rGO and MPc. Therefore, the significantly enhanced electron transfer, electrical conductivity, and gas adsorption due to the combination of rGO and MPcs results in the excellent sensing performance of MPc/rGO hybrids.

## Conclusions

In summary, three novel hybrids based on reduced graphene oxide (rGO) and tetra-α-iso-pentyloxyphthalocyanine copper, nickel, and lead (CuPc, NiPc and PbPc) have been successfully fabricated and the ammonia gas-sensitive properties are studied for the first time. FT-IR, UV–vis, XPS, Raman spectra, TEM, and AFM results demonstrate that the MPc molecules were successfully anchored on the surface of rGO sheets through *π*–*π* stacking and form electron transfer interaction from MPc to rGO. MPc/rGO hybrids were explored as gas sensors and exhibit improved sensing performances to NH_3_ gases at room temperature in comparison to that of pure rGO. The MPc/rGO hybrids exhibited high response value, fast recovery behavior, good reproducibility, selectivity, and stability to NH_3_ gases. The enhanced sensing properties are attributed to the synergistic effect of MPc and rGO in the hybrids with strong electron transfer interaction, superior electrical conductivity, and gas adsorption activity. Strategies for combining various MPcs and nanoscale rGO will open new opportunities for designing and developing low power, low cost, and portable gas sensors.
